# Paraspinal Muscle Segmentation Based on Deep Neural Network

**DOI:** 10.3390/s19122650

**Published:** 2019-06-12

**Authors:** Haixing Li, Haibo Luo, Yunpeng Liu

**Affiliations:** 1 Shenyang Institute of Automation, Chinese Academy of Sciences, Shenyang 110016, China; luohb@sia.cn (H.L.); ypliu@sia.cn (Y.L.); 2 Institutes for Robotics and Intelligent Manufacturing, Chinese Academy of Sciences, Shenyang 110016, China; 3 University of Chinese Academy of Sciences, Beijing 100049, China; 4 Key Laboratory of Opto-Electronic Information Processing, Chinese Academy of Science, Shenyang 110016, China; 5 The Key Lab of Image Understanding and Computer Vision, Liaoning province, Shenyang 110016, China

**Keywords:** paraspinal muscles, segmentation, U-Net, residual module, FPA module

## Abstract

The accurate segmentation of the paraspinal muscle in Magnetic Resonance (MR) images is a critical step in the automated analysis of lumbar diseases such as chronic low back pain, disc herniation and lumbar spinal stenosis. However, the automatic segmentation of multifidus and erector spinae has not yet been achieved due to three unusual challenges: (1) the muscle boundary is unclear; (2) the gray histogram distribution of the target overlaps with the background; (3) the intra- and inter-patient shape is variable. We propose to tackle the problem of the automatic segmentation of paravertebral muscles using a deformed U-net consisting of two main modules: the residual module and the feature pyramid attention (FPA) module. The residual module can directly return the gradient while preserving the details of the image to make the model easier to train. The FPA module fuses different scales of context information and provides useful salient features for high-level feature maps. In this paper, 120 cases were used for experiments, which were provided and labeled by the spine surgery department of Shengjing Hospital of China Medical University. The experimental results show that the model can achieve higher predictive capability. The dice coefficient of the multifidus is as high as 0.949, and the Hausdorff distance is 4.62 mm. The dice coefficient of the erector spinae is 0.913 and the Hausdorff distance is 7.89 mm. The work of this paper will contribute to the development of an automatic measurement system for paraspinal muscles, which is of great significance for the treatment of spinal diseases.

## 1. Introduction

**Figure 1:**
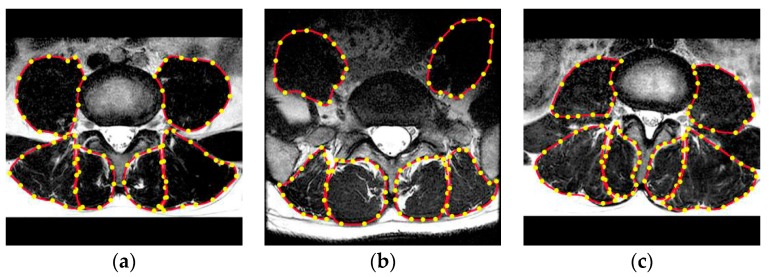
(**a**–**c**) Experts manually construct the outer edge polygon points (yellow) around each muscle. The area enclosed by the red curve connected by the yellow dots indicates the cross-sectional area (CSA) of each muscle.

The paraspinal muscle (multifidus and erector spinae) in particular is important for the dynamic stability of the spine [1]. Evidence suggests that paraspinal muscle atrophy and fatty infiltration occur in patients with chronic low back pain (LBP), disc herniation and lumbar spinal stenosis (LSS) [2]. The standard parameters used to evaluate paraspinal muscle include the cross-sectional area (CSA) and fat infiltration rate [3,4,5,6]. At present, the CSA is measured by tracing the outer fascial boundaries of each muscle using computer software (shown in Figure 1), followed by calculating the rate of high-intensity areas within muscle as the fat infiltration ratio, using a pseudo-coloring technique or histographic analysis [3,6]. However, manual annotation is a time-consuming and laborious task even for experienced radiologists. In addition, manual annotation often suffers large intra- and inter-observer variability, which affects the quality of the paraspinal muscle analysis. Therefore, in order to minimize the workload of experts in reading slices and improve the accuracy of the annotation process, an automatic and reliable paraspinal muscle segmentation method for MR images is urgently needed.

**Figure 2:**
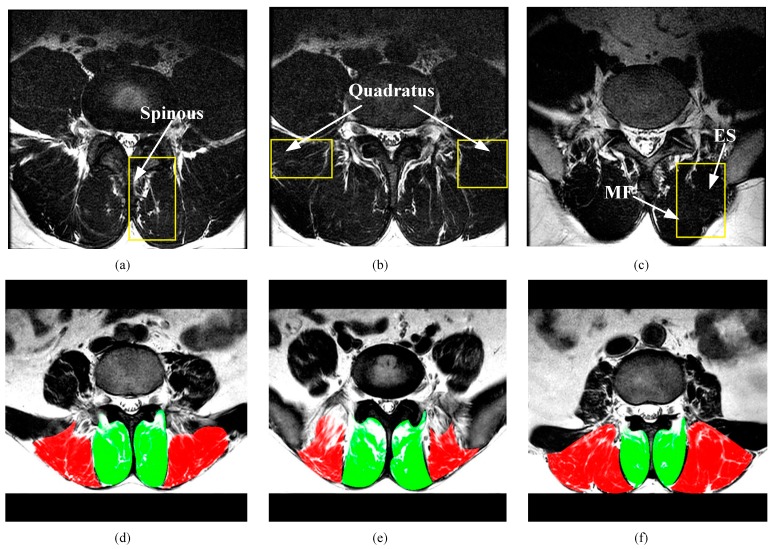
Typical paraspinal muscle Magnetic Resonance (MR) images and their difficulty in segmentation. (**a**–**c**) show images of three patients whose target muscles are unclear. Yellow rectangular boxes indicate the area that is easily segmented incorrectly. The spinous processes in (**a**), the quadratus in (**b**), and the unobvious boundaries between MF and ES in (**c**) effect the segmentation of the target. (**d**–**f**) show three slices from different spinal levels in the same patient. Green denotes the MF muscle and red denotes the ES muscle. Both the MF muscle and the ES muscle have a pronounced deformation.

There are some technical challenges to accurately and automatically segmenting the paraspinal muscle in MR images: (1) the boundary between the target and surrounding structures is often unclear, especially when the multifidus (MF) muscle is close to the erector spinae (ES) muscle. As shown in Figure 2a–c, the boundaries are difficult to distinguish even after careful contrast adjustment; (2) the MF muscle and the ES muscle have a similar gray distribution to the background; (3) the shape of the paraspinal muscles is highly variable, and there are significant changes between patients and even every spinal level of the patient, as shown in Figure 2d–f. 

Due to the above challenges, there is no computer-assisted automatic segmentation method for paraspinal muscle. Most medical research institutions still use manual or semi-automatic methods to segment paraspinal muscle. In 2004, Craig A. et al. used image analysis software to manually segment the lean paraspinal muscle, vertebral body bone and intermuscular fat [7]. In 2009, Craig M. developed a statistical shape modeling to segment the lumbar quadratus [8]. Hu et al. traced the region of interest (ROI) twice within 3 weeks in 29 patients with chronic low back pain to manually measure the functional cross-sectional area and calculate the intra- and interobserver reliability in 2010 [9]. Maryse et al. quantified the composition of the multifidus muscles’ MRI with a semi-automated threshold algorithm in 2017 [10]. Rebecca et al. manually defined the ROI in the quantitative analysis of paraspinal muscle fatty infiltration in 2017 [11]. In 2017, Ryan J. et al. have developed a method for segmenting five bilateral cervical muscles and the spine via ultrasound alone, in real time [12]. In 2018, David et al. used two methods to manually define the ROI of the paraspinal muscles and verified the reliability of the methods [13]. Xiao et al. constructed population-averaged MRI atlases for the image processing and assessment of the lumbar paraspinal muscles in 2018 [14]. However, as the number of processed images increases, the intra-reliability and inter-reliability of the manually segmented image is reduced, and accurate segmentation results cannot be provided.

Recently, convolutional neural networks, which can be trained end-to-end, have made great progress in semantic segmentation and have become the technology of choice in computer vision. Further, the full convolution network (FCN) [15] can be effectively applied to an entire input image, improving learning efficiency. The medical image analysis community has taken notice of these pivotal developments. For medical image analysis, the most successful application is U-Net [16], which is designed with contractive and expansive U-paths. FCN and U-Net have been widely used in cardiac and brain MR images [17,18,19,20,21,22] and have significantly promoted the advancement of automatic medical image segmentation and more complex medical morphology analysis techniques. However, the direct use of these FCNs to segment paraspinal muscle in MR images does not generate good results for the following reasons. First, these networks do not have an effective mechanism to address the challenges of the unclear boundary and large shape changes in the paraspinal muscle segmentation. Second, the purpose of these networks is to label each pixel belonging to the target muscle and therefore not make full use of meaningful contextual information. Finally, the entire spine MR image introduces a complex background for the target muscle segmentation, making these networks difficult to optimize. To this end, we propose a segmentation framework with a residual module [23] and FPA module [24] to complete the challenging paraspinal muscle segmentation. Specifically, the convolutional layer in the U-shaped path is replaced with a residual module to preserve more original information. Then, an FPA module is added after feature extraction, which gradually integrates the feature information at different scales so that the context features of adjacent scales can be more accurately combined and to provide better pixel-level attention for the high-level feature map.

The remainder of this paper is structured as follows: Section 2 presents the method proposed in this paper, the experimental results and discussion are given Section 3, and finally the conclusion can be found in Section 4.

## 2. Methods

**Figure 3:**
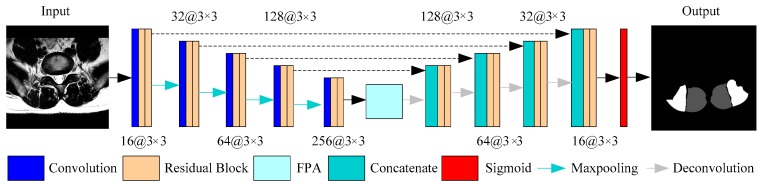
An illustration of the proposed framework for multifidus (MF) and erector spinae (ES) segmentation in MR images.

In this section, we will describe the segmentation framework of the MF and ES muscle in detail. The whole architecture of the framework is shown in Figure 3. We use a U-Net-like structure as the backbone because it performs excellently in medical image segmentation. We have mainly made two improvements: the residual blocks replace the partial convolution layers in the network, which strengthens the details of the image, while the purpose of the feature pyramid attention module is to focus on the paraspinal muscle features while suppressing irrelevant background information.

### 2.1. Preprocessing

**Figure 4:**
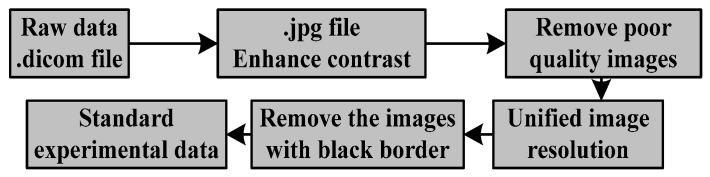
The flowchart of preprocessing.

As shown in Figure 4, the first step in the segmentation of paraspinal muscles is preprocessing. The images were collected and labeled by the spine surgery department of Shengjing Hospital of China Medical University. In order to facilitate the follow-up experiment, we first converted the .dicom file taken by MRI technology into the common .jpg format and enhanced the contrast of the images to make the images easier to observe. The irregular movement of the patient in shoots can cause blurred images and missed muscle information, which will bring challenges to automatic segmentation. Therefore, we eliminate this type of image. In addition, we artificially unified the resolution to 512 × 512 pixels by using a resize function, because the images come from different periods and different equipment. In order to make the data used in the experiment more reliable, we will also remove the images in which the target is in contact with the black border. Finally, we obtain reliable data for training and testing through the above processing.

### 2.2. Residual Module

**Figure 5:**
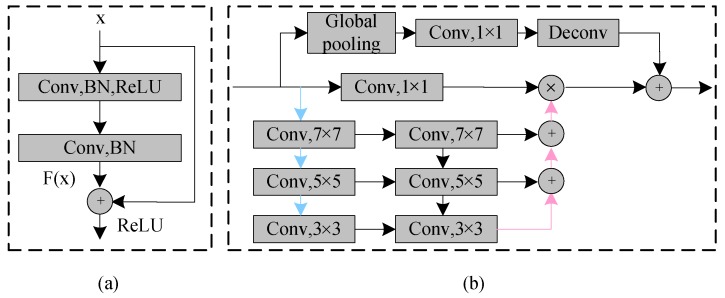
(**a**) Residual block. (**b**) Feature pyramid attention (FPA) module. The blue and pink lines represent the down-sample and upsample operators, respectively.

The residual block was proposed by He Kaiming et al. in 2015. The use of the residual block solves the degradation problem caused by increasing the network depth, which can improve the network performance by simply increasing the network depth. In the traditional convolutional neural network, each layer will have a certain loss of information after passing through the convolution kernel. The residual structure unit adds a "shortcut connection" in the design, which combines the clearer vector data of the upper layer and the convolutional data as the input of the next layer, thus retaining more abundant original information. As shown in Figure 5a, the output of the residual block is added by the input and the output of two concatenated convolutions, and then activated by Rectified Linear Unit (ReLU). In forward propagation, the residual output expression is the accumulation of input *x* and *F*(*x*) for a layer, while the output of a traditional neural network is a cumulative multiplication expression. Therefore, in back-propagation, the gradient of the residual network is accumulated and the gradient of the traditional network is the multiplication, and the multiplication causes the gradient to disappear inevitably, while the residual network can avoid the gradient disappearing and solve the gradient of the extremely deep network and make the deep network possible. In this paper, we use a two-layer residual learning unit that contains two 3 × 3 convolutions of the same number of output channels and a bypass, called the shortcut connection. The following is the formula for the residual unit:

(1) y=f(F(x)+x)

where *x* represents the input of a residual block, f(•) is ReLU, F(x) represents the output before the second activation function, and the output of a residual block is *y*.

### 2.3. Feature Pyramid Attention Module

Inspired by the attention mechanism and the spatial pyramid pooling [25], we added an FPA module after four down-samplings. This module not only solves the problem that the attention mechanism cannot effectively extract multi-scale features and lacks the information of the pixel direction, but also solves the problem that the spatial pyramid pooling loses pixel positioning in the pooling operation of different scales. As shown in Figure 5b, the FPA module consists of five parts: the top branch is the global pooling; the remaining branches are the feature maps of different scales (32 × 32, 16 × 16, 8 × 8, 4 × 4) from top to bottom. The FPA module can fuse context information of different scales, increase the pixel-level receptive field and provide better pixel-level attention for high-level feature maps. There are three down-sampling branches in this structure, each of which uses 7 × 7, 5 × 5, 3 × 3 convolution kernels to extract features of different scales. Because of the low resolution of high-level feature maps, using a larger kernel does not entail too much computational burden. Subsequently, the pyramid structure gradually integrates the feature information at different scales, so that the context features of adjacent scales can be more accurately combined. The original features of the network are then multiplied pixel by pixel by a 1 × 1 convolution. Finally, the global pooling branch is introduced to concentrate the output features, which further improves the performance of the FPA module.

### 2.4. Network Architecture

We implemented a slightly modified version of the U-Net architecture shown in Figure 3 with the residual module and the feature pyramid attention module. Our implementation differs from the original U-Net, which has about 28 million (M) parameters. The parameters of U-Net can be reduced by adjusting the depth of model or the channels of convolution layer, so that the U-Net model can be lightweight compared to other models. We decreased the channels to the original 1/4 that reduced the model parameter size to 2.53M. The purpose of this is because the number of medical images is less than that of natural images, and it is more difficult to obtain clinically meaningful labels. Therefore, the network model for medical images should not be too complicated, and there should not be too many parameters. In detail, we extract the feature maps with the ResNet-18 structure, and the size of the output feature map is 1/16th of the input image. The FPA module is the central block between the encoder and decoder structure, which can gather dense pixel-level attention information from the feature maps of the Resnet-18. Combined with the global context, the high-level features and the low-level features are concatenated in an expanding path via skip-connections to generate the final predicted map.

## 3. Experiment and Results

### 3.1. Dataset

All data were from the same hospital and comprised young male patients in northern China, aged 18 to 35 years. The raw data of such patients is relatively uniform, characterized by relatively advanced paraspinal muscles, a lower degree of fat, and clear boundaries. Philips magnetic resonance was used, and the repetition time of sagittal scanning was 2500 ms and that of axial scanning was 24855 ms; the echo time of sagittal was 80 ms, that of axial scanning was 120 ms and that of axial scanning was 4 mm under 3.0 T. All the patients’ lumbar MR scans included T2 weighted images. The sagittal position nearest to the midline was selected as the location image. The axial images corresponding to L3-4, L4-5 and L5-S1 discs were scanned. Each disc was divided into three slices. By excluding obvious disc herniation, infection, fracture, tumor and other abnormal changes and incomplete images, 1080 T2-weighted axial images of 120 patients were obtained. All images are processed by brightness and contrast adjustment and normalized operation. Four spine surgeons and two imaging surgeons used Photoshop graphics software to label the bilateral erector spine muscles and multifidus muscles in the image manually, which were double-checked by one spine surgery specialist with more than 30 years of experience and two spine surgeons with more than 10 years of experience.

### 3.2. Implementation Details

Our network was implemented by Python 2.7 and Keras 2.2.4, and our model was trained and tested on a Nvidia GeForce GTX TITAN X GPU, developed on a 64-bit ubuntu 14.04 platform with Intel Core i7-5930K CPU with 64 GB RAM. Five-fold cross-validation is employed for comparison with the state-of-the-art methods. The original dataset is randomly split into five equal size folds. In each round, a single fold is retained as the testing data for testing the model, and the remaining four folds are used for training. We record the model at each training epoch, and the model that performs best is used to evaluate the performance. The five results from the folds can then be averaged to produce a single result. The proposed model directly handles paraspinal MRIs without any post-processing and data augmentation. The model is based on the stochastic gradient descent method (SGD) for optimization. A momentum coefficient is 0.9 and the initial learning rate is 0.1. The learning rate varies with the training epochs, when training epochs is 60, the decay argument is specified, decay=0.1/epochs, and the learning rate of each training is decreased to lr=lr/(1+decay×epoch). We randomly initialize parameter weights according to the Xavier scheme. The batch size is set to 4 because of GPU limitations. The loss function is the negative Dice coefficient [26]:

(2) $$Loss=-\frac{2\sum_{i}^{N}p_{i}g_{i}}{\sum_{i}^{N}p_{i}+\sum_{i}^{N}g_{i}}$$

where $p_{i}$, $g_{i}$ corresponds to the *i*th pixel of the predicted segmentation and ground truth mask, respectively. 

### 3.3. Evaluation Criteria

In order to evaluate the segmentation performance of different methods, we employ the following commonly used medical image segmentation metrics as evaluation criteria. These metrics measure the degree of overlap and also measure the spatial distance, showing the similarities and differences between automatic and manual segmentation.

- Dice similarity coefficient (DSC):
  (3) $$DSC=\frac{2\left\|P_{seg}\cap P_{gt}\right\|}{\left\|P_{seg}\right\|+\left\|P_{gt}\right\|}$$
- True negative rate/specificity (TNR):
  (4) $$TNR=\frac{true\;negative}{false\;positive\;+true\;negative}$$
- True positive rate/sensitivity (TPR):
  (5) $$TPR=\frac{true\;positive}{true\;positive+true\;negative}$$
- Hausdorff distance (HD):
  (6) $$\begin{array}{c} HD\left(P_{gt},P_{seg}\right)=\max(h\left(P_{gt},P_{seg}\right),h\left(P_{seg},P_{gt}\right))\\ h\left(P_{gt},P_{seg}\right)=\underset{a\in P_{gt}}{\max}\underset{b\in P_{seg}}{\min}\left\|a-b\right\| \end{array}$$
  where $P_{gt}$ and $P_{seg}$ denote the pixel sets of the manually labeled ground truth and automatically segmented muscle, respectively. DSC measures the overlap of the segmentation with the ground truth, while specificity reflects the miss rate, sensitivity reflects the mistake rate and HD is the maximum distance from all the minimum distances between the boundaries of the ground truth and segmentation. For DSC, TNR, and TPR, the larger the value, the better the performance, while for HD, the smaller the value, the better the performance.

### 3.4. Modules Analysis by Intra-Comparison

**Figure 6:**
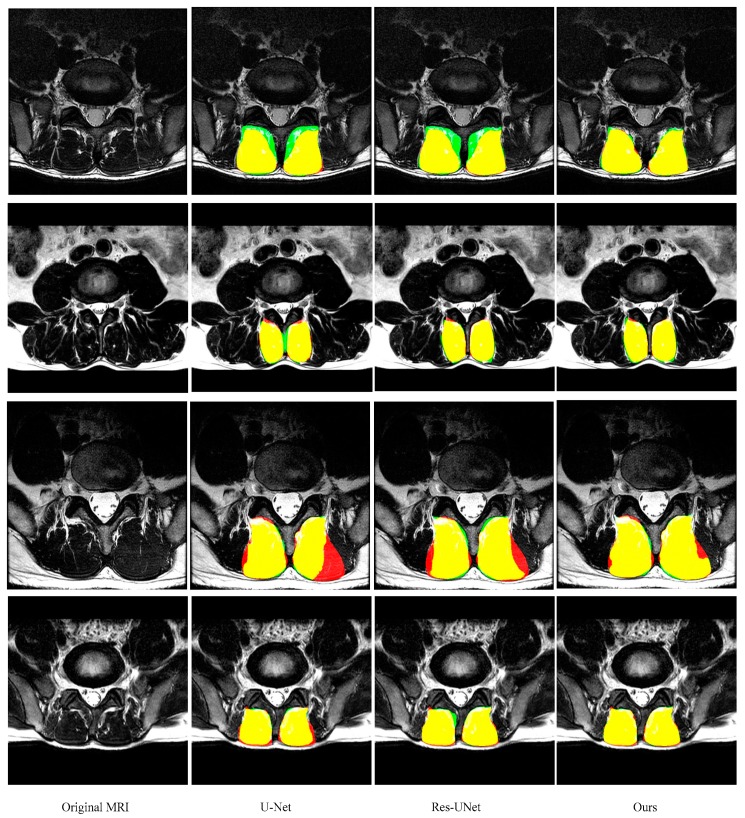
Representative cases of the segmentation results of the MF (green is predicted segmentation, red is ground truth mask and yellow is the overlap region) obtained by U-Net, ResU-Net, and our segmentation network. These images are from different patients and shown in the axial view.

**Figure 7:**
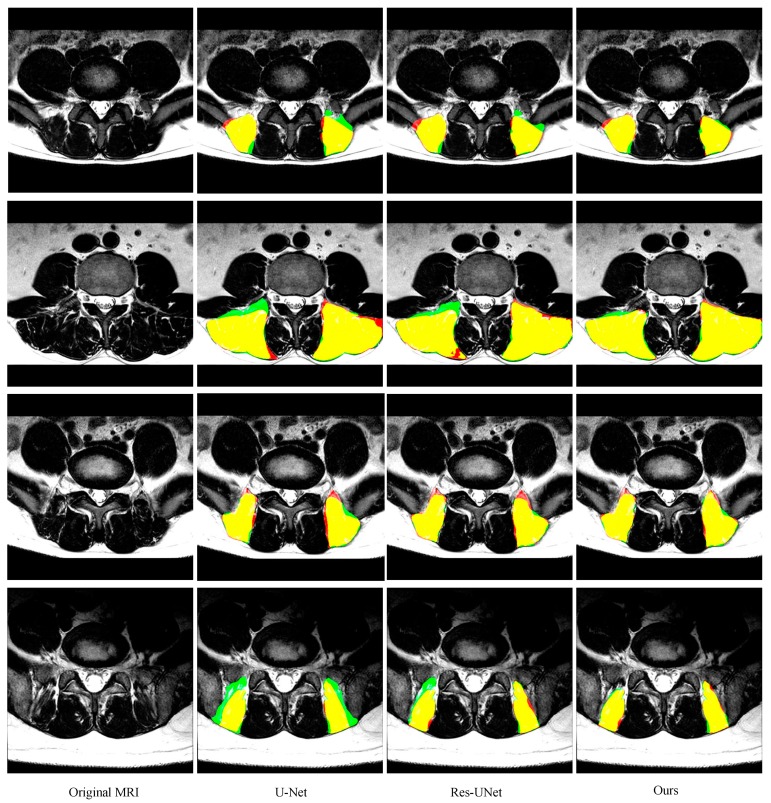
Representative cases of the segmentation results of the ES obtained by U-Net, ResU-Net, and our segmentation network.

As shown in Figure 6 and Figure 7 and Table 1 and Table 2 (from row 5 to row 7), two improvements in this paper give the model superior performance in the segmentation of the paraspinal MF and ES. As a baseline, U-Net on average achieves 0.921 ± 0.039 DSC, 0.925 ± 0.049 sensitivity, 0.920 ± 0.056 specificity and 6.16 ± 5.14 mm HD for the MF segmentation. The DSC, sensitivity, specificity and HD of the ES segmentation are 0.895 ± 0.080, 0.917 ± 0.086, 0.887 ± 0.105 and 9.75 ± 8.72, respectively. After adding the residual module, the false segmentation of the muscle has been improved to some extent. This not only proves the effectiveness of the residual module, but also proves that the residual module can obtain more target details. Although ResU-net can improve the performance of the traditional U-Net, it cannot solve the interference of other tissues on the target muscles (e.g., the adipose tissue between the MF muscle and spinous process in the first MR image of Figure 6; the plaque soft tissue around the ES muscle in the first MR image of Figure 7). The use of the FPA module has significantly improved the segmentation performance. The module preserves the fine-grained detailed differences between the target muscle and other tissues by combining with the global context. In addition, the FPA module has better adaptability to highly deformed muscle tissue. As shown in the 3rd row of Figure 6 and the 4th row of Figure 7, U-Net and ResU-net only learned part of the muscle morphology and thus got poor results, while the addition of the FPA module made the model more focused on the diversity of muscle morphology and obtained better results in segmentation.

### 3.5. Comparison with other State-of-the-Art Methods

We compare our method to the performance of other existing segmentation methods on the same dataset as shown in Table 1 and Table 2 (from the 1st row to the 4th row). FCN predicts each pixel almost independently of each other, which leads to a lack of spatial continuity. Our method significantly outperforms FCN in all four evaluation metrics. The segmentation performance of PSPNet [26] and SegNet [27] is better than FCN, but these two methods lose pixel localization during different scale pooling operations. The atrous spatial pyramid pooling module in Deeplabv3+ [28] may cause local information loss, which is harmful for the local consistency of the feature map. Compared to other networks, our method has fewer parameters (shown in Table 3), which effectively reduces the test time. Specifically, our method has only 5M parameters, while FCN has 11.2M parameters, SegNet has 29.4M parameters, PSPNet has 11.2M parameters, and deeplabv3+ has up to 41M parameters. Therefore, our method has strong predictive performance and application ability in the measurement of paraspinal muscle.

### 3.6. Muscle CSA Measurements

Because most lumbar diseases and muscle morphological changes occurred in L3-4, L4-5 and L5-S1 discs, in the last part of the study, 216 test images of the bilateral total cross-sectional area of MF and ES muscles were measured. Muscle measurement first converts the manually labeled closed curve to a mask by using a thresholding and hole-filling method, and then evaluates the same MRI slice using an automated algorithm. After training the proposed network, we fed-back each slice to obtain an output image with a pixel value as the likelihood of being part of paraspinal muscle. By binarizing and averaging all pixel values, we can obtain the area fraction of paraspinal muscle in this slice. The physical area of the paraspinal muscle can be derived under the premise of knowing the pixel resolution and image size. The whole process is as follows:

(7a) $$P=f_{net}(I)$$

(7b) B=binarize(P)

(7c) F=mean(B)

(7d) $$A=F{\left(wL\right)}^{2}$$

where *I* is the input image, and *P* is the output image whose value is the probability of the paraspinal muscle. *B* is the binarized output image, *F* is the area fraction, w is the physical length of the pixel, *L* is the image length, and *A* is the physical area of the paraspinal muscle.

We use a linear regression curve to show the correlation of the CSA obtained by manual segmentation and automatic segmentation. The linear regression equation is calculated as follows:

(8) $$y=b_{0}+b_{1}x$$

where *x* represents the predicted area, *y* stands for true area, and $b_{0}$ and $b_{1}$ represent the relationship between *x* and *y*.

(9a) $$b_{1}=\frac{\sum \left(x_{i}-\bar{x})(y_{i}-\bar{y}\right)}{\sum {\left(x_{i}-\bar{x}\right)}^{2}}$$

(9b) $$b_{0}=\bar{y}-b_{1}\bar{x}$$

where $\bar{x}$ is the mean of the area of predicted images, and $\bar{y}$ is the mean of the area of ground truth. The closer the regression curve is to y=x, the closer the predicted area is to the ground truth.

**Figure 8:**
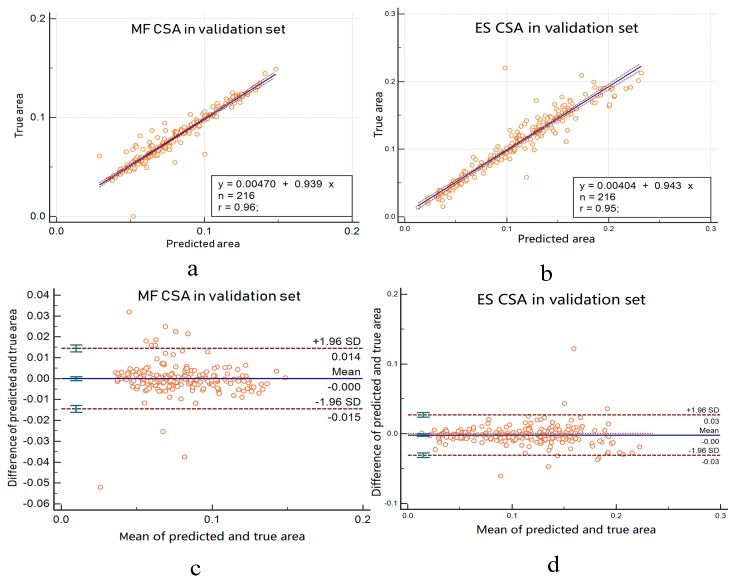
(**a**,**b**) Linear regression for multifidus and erector spine CSA. The blue curve is the regression curve, the orange circle is the predicted area for each image, the value of *n* is the number of test samples, and the value of *r* is R squared. (**c**,**d**) Bland–Altman analysis for multifidus and erector spinae CSA.

We also calculate R squares when calculating the regression curve. This value is called the judgment coefficient and is used to measure the goodness of fit of the regression equation. The larger the value, the more meaningful the regression equation is, and the higher the interpretation of the dependent variable by the independent variable. In Figure 8a,b, the regression curves are y=0.047+0.939x and y=0.004+0.943x, respectively. This curve demonstrates the overlap between automated and manual area is large. The R squares are 0.96 and 0.95, respectively. This result indicates that the cross-sectional area of the automatic segmentation is highly correlated with the manually calibrated cross-sectional area. 

Figure 8c,d gives the Bland–Altman analysis about the automated area from the bilateral multifdus and erector spinae and manually obtained area. Two methods are considered to have good agreement when the measurement difference is small enough for both methods to be used interchangeably [29,30]. In accordance with Bland–Altman, all the plots show good agreement between the manual labels and automated method and no systematic bias; the distribution of the scores around the mean approximates zero and is spread evenly and randomly above and below the line. A histogram of the difference scores was also prepared for every measurement parameter, and all histograms followed a normal distribution. As such, because the error is normally distributed, we can observe that about 95% of the points are between the limits of agreement for each measure. The width of the limits of agreement is also small.

## 4. Conclusions

In this study, we improved a method of the automatic segmentation of lumbar paraspinal muscle MR images based on deep convolutional neural networks. The automatic segmentation results visually show good agreement with the manually labeled ground truth, indicating that the proposed method has the same potential as a doctor to distinguish between different paraspinal muscle MR images. The experimental results show that the method has better segmentation visualization and quantitative evaluation. Quantitative assessment yielded better results than other automated segmentation algorithms, with DSC, sensitivity, specificity and HD indicators reaching 0.949 ± 0.034, 0.951 ± 0.046, 0.950 ± 0.035 and 4.62 ± 2.81 (0.913 ± 0.08, 0.920 ± 0.100, 0.919 ± 0.073, 7.89 ± 5.61), indicating that the method can provide a reference for radiologists. However, there is a problem that needs to be improved when we use five-fold cross-validation method. We should add another test set to ensure the fairness of the evaluation results. The proposed method can quickly calculate the cross-sectional area of the paraspinal muscles, which provides a convenient condition for doctors to screen sarcopenia and also quantify the changes of paraspinal muscles before and after lumbar spine surgery. In the future, our algorithm should be prospectively evaluated in a larger database, including the quantitative cross-sectional area of functional paraspinal muscles and the degree of fat infiltration in different genders and age groups. 

## Figures and Tables

**Table 1:** The performance of various models on the MF test set (the best results are indicated in bold).

Method	DSC	Sensitivity	Specificity	HD (mm)
FCN	0.908 ± 0.057	0.925 ± 0.069	0.878 ± 0.057	10.76 ± 10.0
SegNet	0.938 ± 0.038	0.949 ± 0.472	0.930 ± 0.052	7.51 ± 8.29
PSPNet	0.936 ± 0.036	0.931 ± 0.043	0.944 ± 0.053	5.19 ± 3.84
DeepLabv3+	0.943 ± 0.035	0.940 ± 0.042	0.947 ± 0.044	5.02 ± 3.89
U-Net	0.921 ± 0.039	0.925 ± 0.049	0.920 ± 0.056	6.16 ± 5.14
ResU-Net	0.944 ± 0.043	0.946 ± 0.063	0.945 ± 0.045	4.68 ± 3.25
**Ours**	**0.949** ± **0.034**	**0.951** ± **0.046**	**0.950** ± **0.035**	**4.62** ± **2.81**

**Table 2:** The performance of various models on the ES test set (the best results are indicated in bold).

Method	DSC	Sensitivity	Specificity	HD (mm)
FCN	0.873 ± 0.079	0.865 ± 0.075	0.892 ± 0.111	15.24 ± 14.85
SegNet	0.904 ± 0.082	0.918 ± 0.096	0.901 ± 0.092	9.9 ± 9.85
PSPNet	0.901 ± 0.081	0.90.1 ±0.089	0.915 ± 0.098	8.46 ± 6.55
DeepLabv3+	0.908 ± 0.077	0.919 ± 0.075	0.908 ± 0.10	8.19 ± 5.92
U-Net	0.895 ± 0.080	0.917 ± 0.086	0.887 ± 0.105	9.75 ± 8.72
ResU-Net	0.905 ± 0.092	0.915 ± 0.102	0.902 ± 0.109	8.86 ± 8.42
**Ours**	**0.913** ± **0.082**	**0.920** ± **0.100**	**0.919** ± **0.073**	**7.89** ± **5.61**

**Table 3:** The parameters of various models (the model with the fewest parameters is indicated in bold).

Method	FCN	SegNet	PSPNet	DeepLabv3+	U-Net	ResU-Net	Ours
Parameter	10.9M	29.4M	11.2M	41M	28.8M	5.1M	**5.0M**
